# Enhancing Mental and Physical Health of Women through Engagement and Retention (EMPOWER) 2.0 QUERI: study protocol for a cluster-randomized hybrid type 3 effectiveness-implementation trial

**DOI:** 10.1186/s43058-022-00389-w

**Published:** 2023-03-08

**Authors:** Alison B. Hamilton, Erin P. Finley, Bevanne Bean-Mayberry, Ariel Lang, Sally G. Haskell, Tannaz Moin, Melissa M. Farmer, the EMPOWER QUERI Team

**Affiliations:** 1 Veterans Affairs Health Services Research and Development (HSR&D) Center for the Study of Healthcare Innovation, Implementation & Policy, VA Greater Los Angeles Healthcare System, Los Angeles, CA USA; 2 Department of Psychiatry and Biobehavioral Sciences, David Geffen School of Medicine, University of California Los Angeles, Los Angeles, CA USA; 3 Departments of Medicine and Psychiatry and Behavioral Sciences, University of Texas Health Science Center, San Antonio, TX USA; 4 Division of General Internal Medicine, David Geffen School of Medicine, University of California Los Angeles, Los Angeles, CA USA; 5 VA San Diego Healthcare System, San Diego, CA USA; 6 Department of Psychiatry, University of California San Diego, San Diego, CA USA; 7 VA HSR&D Pain Research, Informatics, Multi-morbidities, and Education Center, VA Connecticut Healthcare System, West Haven, CT USA; 8 Department of Internal Medicine, Yale School of Medicine, Yale University, New Haven, CT USA; 9 Division of Endocrinology, Diabetes & Metabolism, David Geffen School of Medicine, University of California Los Angeles, Los Angeles, CA USA

**Keywords:** Hybrid effectiveness-implementation trial, Mixed methods, Evaluation, Implementation strategies, Replicating effective programs, Evidence-based quality improvement, Diabetes Prevention Program, Telephone Lifestyle Coaching, Reach Out, Stay Strong Essentials, Stages of Implementation Completion

## Abstract

**Background:**

Women Veterans are the fastest-growing segment of Veterans Health Administration (VA) users. The VA has invested heavily in delivering care for women Veterans that is effective, comprehensive, and gender-tailored. However, gender disparities persist in cardiovascular (CV) and diabetes risk factor control, and the rate of perinatal depression among women Veterans is higher than that among civilian women. Challenges such as distance, rurality, negative perception of VA, discrimination (e.g., toward sexual and/or gender minority individuals), and harassment on VA grounds can further impede women’s regular use of VA care. Enhancing Mental and Physical Health of Women through Engagement and Retention (EMPOWER) 2.0 builds on work to date by expanding access to evidence-based, telehealth preventive and mental health services for women Veterans with high-priority health conditions in rural and urban-isolation areas.

**Methods:**

EMPOWER 2.0 will evaluate two implementation strategies, Replicating Effective Practices (REP) and Evidence-Based Quality Improvement (EBQI), in supporting the implementation and sustainment of three evidence-based interventions (Virtual Diabetes Prevention Program; Telephone Lifestyle Coaching Program; and Reach Out, Stay Strong Essentials) focused on preventive and mental health care for women Veterans. We will conduct a mixed-methods implementation evaluation using a cluster-randomized hybrid type 3 effectiveness-implementation trial design to compare the effectiveness of REP and EBQI on improved access to and rates of engagement in telehealth preventive lifestyle and mental health services. Other outcomes of interest include (a) VA performance metrics for telehealth care delivery and related clinical outcomes; (b) progression along the Stages of Implementation Completion; (c) adaptation, sensemaking, and experiences of implementation among multilevel stakeholders; and (d) cost and return on investment. We will also generate implementation playbooks for program partners to support scale-up and spread of these and future evidence-based women’s health programs and policies.

**Discussion:**

EMPOWER 2.0 provides a model for mixed-methods hybrid type 3 effectiveness-implementation trial design incorporating evaluation of performance metrics, implementation progress, stakeholder experience, and cost and return on investment, with the ultimate goal of improving access to evidence-based preventive and mental telehealth services for women Veterans with high-priority health conditions.

**Trial registration:**

ClinicalTrials.gov, NCT05050266. Registered on 20 September 2021.

**Supplementary Information:**

The online version contains supplementary material available at 10.1186/s43058-022-00389-w.

Contributions to the literature

- Relatively few studies have compared the effectiveness of implementation strategies for achieving the rollout of evidence-based interventions across large healthcare systems.
- Conducting a rigorous comparison of clinical benefit, cost and return on investment, and time to achieving implementation associated with two different implementation strategies—Replicating Effective Programs (REP) and Evidence-Based Quality Improvement (EBQI)—can directly support evidence-based policy and resource allocation decisions.
- This study protocol provides a model for mixed-method implementation evaluation in a large healthcare system.

## Background

As the fastest-growing segment of users in the Veterans Health Administration (VA) [1], the number of women Veterans receiving care in the VA is expected to increase by 73%, from 9.3 to 16.4%, between 2015 and 2043 [2]. In anticipation of this increase, the VA has invested heavily in providing effective, comprehensive, and gender-tailored care [3, 4] to address women Veteran patients’ unique and complex healthcare needs [5–7]. However, gender disparities persist in cardiovascular (CV) and diabetes risk factor control in and out of the VA [8, 9]. Also, the prevalence of many mental and physical comorbidities, including perinatal depression, obesity [10], and cardiovascular disease [11], remains higher among women Veterans than among civilian women [12]. Historically, women Veterans have had a high rate of attrition from VA care due to distance and low ratings of VA care quality [13]. Negative perceptions of VA care [14], comorbid mental health issues [15], discrimination based on sexual and/or gender minority identities [16], harassment on VA grounds [17], and distance and rurality [18, 19] are cited barriers in women’s regular use of VA care. Thus, improvements are still needed to increase women Veterans’ access to and engagement in convenient, safe, evidence-based, patient-centered care that achieves the VA “lane of effort” of Veterans’ “lifelong health, well-being, and resilience” [20].

Telehealth, or the delivery of healthcare via remote technologies including telephone, video calls, and online, is ideal for addressing existing gaps in care for women Veterans. Barriers to gender-specific care include the low numbers of women Veterans at any single VA location, access barriers due to work, travel times, and school and caregiving responsibilities, particularly for women Veterans living in rural areas. Telehealth can address these barriers by providing flexible, individualized care and reducing travel burden. The evidence suggests that women Veterans stand to benefit from telehealth approaches, particularly in healthy lifestyle change for disease prevention (including increasing physical activity, weight management, smoking cessation, and diet) and reproductive health (including contraception, prenatal care, maternal mental health, lactation, and postpartum care) [21].

Since its inception in 2015, the *E*nhancing *M*ental and *P*hysical health *o*f *W*omen through *E*ngagement and *R*etention (EMPOWER) QUERI 1.0 team has focused on implementing gender-tailored, preference-based care models for women Veteran patients with high-priority health conditions: (1) type 2 diabetes risk, (2) cardiovascular risk, and (3) anxiety and/or depression [22]. In EMPOWER 1.0 studies, women Veterans expressed preferences for gender-specific (women only) care and for telehealth care options [23]. Therefore, we are extending our overarching commitment to improving women Veterans’ engagement and retention in care with our EMPOWER 2.0 Impact Goal to expand access to evidence-based, preventive lifestyle and mental health services delivered by telehealth for women Veterans with high-priority health conditions in rural and urban-isolation areas. To achieve this impact goal, we propose a coordinated program with the following aims:

1. Using two implementation strategies (Replicating Effective Practices [REP] [24] and Evidence-Based Quality Improvement [EBQI] [25]), support implementation and sustainment of three evidence-based practices (EBPs) focused on preventive lifestyle and mental health care for women Veterans across 20 VA facilities (10 with REP, 10 with EBQI), several of which are rural, low-performing in women’s health care, and/or lead sites for high-reliability organization [20].
2. Conduct a mixed methods implementation evaluation using a cluster randomized hybrid type 3 effectiveness-implementation trial design [26]. We will compare the effectiveness of REP and EBQI in improved access to and rates of engagement in preventive lifestyle and mental telehealth services. Other outcomes include (a) VA performance metrics for telehealth care delivery and related clinical outcomes for women Veterans; (b) progression along the *Stages of Implementation Completion* [27]; (c) adaptation, sensemaking [28], and experiences of EBP implementation among multilevel stakeholders [29]; and (d) cost and return on investment [30].
3. Generate implementation “playbooks” [22] for program partners that are scalable and serve as guidance for future implementation of a broader array of evidence-based women’s health programs and policies.

These conceptually and methodologically linked aims will be realized by an experienced multidisciplinary team with a strong track record of collaboration and guided by a Technical Expert Panel of operational and research partners and subject matter experts. We will implement three EBPs: (1) *Virtual Diabetes Prevention Program (Virtual DPP)* [31], an evidence-based lifestyle intervention, emphasizing moderate weight loss, diet, and physical activity, shown to prevent and/or delay progression to type 2 diabetes; (2) *Telephone Lifestyle Coaching Program (TLC)* [32], developed by one of our partners (National Center for Disease Prevention and Health Promotion), which provides evidence-based, individual-level, personalized health coaching focused on wellness and cardiovascular disease prevention; and (3) *Reach Out, stay Strong, Essentials (ROSE)* [33], an evidence-based intervention for prevention of perinatal depression that can be delivered via telehealth. (For detailed background on these EBPs and related health conditions among women Veterans, see Additional file 1.)

## Methods/Design

### Overview of study design

In this 5-year study, we will conduct a cluster randomized hybrid [26] type 3 effectiveness-implementation trial to evaluate the effectiveness of two implementation strategies (REP and EBQI) in supporting the implementation of three evidence-based practices (virtual DPP, TLC, and ROSE) to increase use of prevention-focused telehealth services among women Veterans (Aim 1). Below we describe our three Aims in detail.

### Aim 1: Using two implementation strategies (REP [24] and EBQI [25]), support implementation and sustainment of three EBPs focused on preventive lifestyle and mental health care for women Veterans across 20 VA facilities (10 REP and 10 EBQI sites)

#### Implementation strategies

EMPOWER 2.0 focuses on comparing two evidence-based implementation strategies described below (Table 1).

**Table 1:** Implementation strategies: key activities by phase

	Years 1-2.5		Years 2.5-4	Year 5
**QUERI Roadmap**	**Pre-implementation** Identify problem & solution Engage stakeholders Develop measures and data		**Implementation** Implement an intervention Activate implementation teams Monitor implementation progress	**Sustainment** Sustain an intervention Transition ownership to stakeholders Ongoing evaluation and reflection
**REP**	***Pre-conditions***	***Pre-implementation***	***Implementation***	***Maintenance and evolution***
	Ensure fit, identify barriers, draft intervention package	Develop package, pilot test, identify champion, hold orientation meetings	Train staff, provide TA, conduct evaluation, measure fidelity, measure outcomes, share results, discuss sustainability	Change practice to facilitate long-term adoption, prepare package for dissemination, recustomize delivery
**EBQI**	Regional stakeholder planning meetings, formative evaluation	Train local QI champions and team members	Provide practice facilitation, expert review and feedback, monthly across-site calls, technical workgroups, formative evaluation	Foster regional spread, summative evaluation

##### Implementation strategy 1—Replicating Effective Programs (REP)

REP has a strong evidence base in VA health services and implementation research—including in EMPOWER QUERI 1.0 [22, 34]—in promoting uptake of evidence-based pratices [24]. Informed by theories of Diffusion of Innovation and Social Learning, the REP framework consists of four phases: pre-conditions, pre-implementation, implementation, and maintenance/evolution [24]. Careful attention is paid to intervention packaging during pre-conditions and pre-implementation; training, technical assistance, and fidelity assessment during implementation; and re-customizing during maintenance/evolution. During each phase, local context is paramount, with varying deployment of the intervention depending on local priorities, needs, and resources. Our team has comprehensive expertise in REP grounded in implementation of three EBPs across diverse sites in EMPOWER 1.0.

##### Implementation strategy 2—Evidence-Based Quality Improvement (EBQI)

Tested in several VA implementation trials (e.g., of collaborative care for depression, VA’s patient-centered medical home (PACT), Women’s Health PACT), EBQI is a systematic QI method for engaging frontline primary care practices in improvement that introduces “best science” and evidence in the service of operational goals and is supported by a partnership between multi-level, interdisciplinary operations stakeholders and a research team [29]. EBQI is aimed at developing learning organizations through multi-level, cross-discipline engagement with science and data. It draws upon explicit QI support by scientific teams to enable context-tailored evidence-based practices; social science theory on provider/team behavior; and improved use of implementation and QI methods. Application of EBQI in the EMPOWER QUERI 2.0 will focus on identifying (with multilevel stakeholders) context-specific design priorities for the three EBPs. Notably, both EMPOWER 2.0 implementation strategies reflect phased approaches consistent with recommendations of the QUERI Implementation Roadmap (Table 1) [35].

We have selected EBQI and REP because they provide differing approaches to supporting sites with implementation of EBPs: EBQI is a higher-intensity strategy that entails multilevel stakeholder engagement, while REP is a lower-intensity strategy with an explicit process framework for local tailoring. Both strategies have strong evidence of effectiveness and are well-established in VA. As noted in the QUERI Roadmap [35], health systems have already invested in EBQI “to scale up and spread their practices and policies nationally,” but we have yet to understand the conditions in which this investment in a high-intensity strategy is warranted, compared to a low-intensity strategy. Comparing these strategies across multiple sites for three different EBPs (virtual DPP, TLC, and ROSE) will provide data on implementation strategy effectiveness and cost, which will be of direct relevance to leadership in decision-making and investment. Findings will allow a detailed examination of how each implementation strategy supported the implementation of each EBP across varying settings, thereby elucidating a blueprint for matching strategies with EBPs in differing organizational contexts.

*Setting and site selection*: We recruited sites (Fig. 2 below) from the Women’s Health Practice-Based Research Network (PBRN), a network that provides a research infrastructure for investigators seeking to increase inclusion of women in VHA research or conduct multi-site women’s focused research in VHA. Comprised of approximately 70 VA sites that see one-half of women Veteran VA users, the PBRN helps investigators overcome the challenges of multi-site studies through the engagement of Site Leads with established working relationships with local clinicians and facility leadership. Sites were also recruited in collaboration with regional leadership in four regional Veterans Integrated Service Networks (VISNs). Each VISN was selected based on leadership’s interest in the EBPs, priorities for women Veterans’ preventive health, and our history of collaboration.

*Low-performing sites*: Our team has a long history of working with sites to improve women Veterans’ health, including QI efforts in low-performing sites. For the current project, a number of our sites are designated low-performing sites based on site visit assessment data.

*Patient eligibility and selection*: Women Veteran VA users are eligible for each EBP as follows: (1) *Virtual DPP*—overweight/obese women Veterans (BMI≥25 kg/m^2^ [≥23 if Asian]) with history of prediabetes (defined as either an HbA1c 5.7-6.5% or fasting blood glucose 100–125 mg/dL or oral glucose tolerance test 140–199 mg/dL) or history of gestational diabetes (GDM) or high risk on diabetes screening questionnaire. Women will receive invitation letters or be referred to the program by a healthcare provider and self-register with a CDC-recognized virtual DPP provider. (2) *TLC*—all women Veterans (who are not pregnant) are eligible to participate in TLC. Women will be referred to the program by a healthcare provider and will receive an outreach call from contracted health coaches to enroll. (3) *ROSE*—All pregnant women Veterans are eligible. Women will be identified and recruited by the site pregnancy/maternity care coordinator or other women’s health staff. As part of each implementation strategy (REP and EBQI, see below), sites will determine their own locally relevant recruitment strategies to engage 40 women Veterans/site in virtual DPP or TLC, and all interested pregnant women in ROSE.

### Aim 2: Conduct a mixed methods implementation evaluation using a cluster-randomized hybrid type 3 effectiveness-implementation trial design comparing the effectiveness of REP and EBQI in terms of (a) improved access to and rates of engagement in preventive lifestyle and mental telehealth services (primary outcome) and improved VA performance metrics for telehealth care delivery and related clinical outcomes for women Veterans; (b) progression along the Stages of Implementation Completion; (c) adaptation, sensemaking, and experiences of EBP implementation among multilevel stakeholders; and (d) cost and return on investment

#### EMPOWER 2.0 Conceptual Framework

**Fig. 1:**
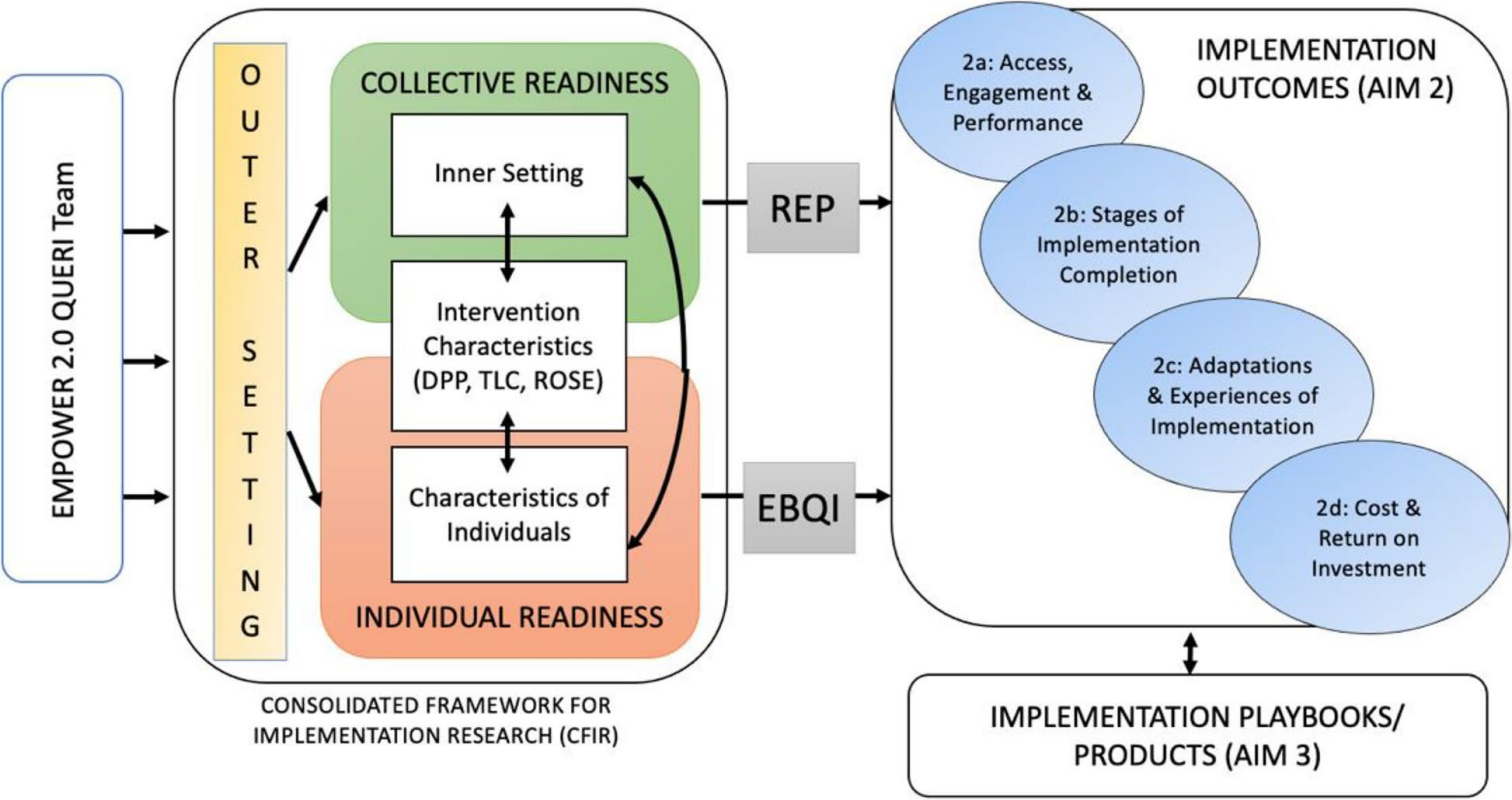
Conceptual model

Figure 1 reflects grounding in the Consolidated Framework for Implementation Research (CFIR) [36] and intention to examine implementation and sustainment outcomes that QUERI and operations partners have indicated are of the highest value. CFIR offers a synthesis of theory and constructs from across implementation science. Constructs are organized into five domains (outer setting, inner setting, characteristics of the intervention, characteristics of individuals, and implementation process), which are dynamic and interrelated in producing implementation readiness at the individual and collective (e.g., clinical unit, facility) levels. CFIR is increasingly used to aid in implementation and evaluation planning and is specifically recommended for use in examining predictive factors associated with implementation outcomes [37]. Because it accounts for individual- and setting-level characteristics and acknowledges how interactions between individuals and their larger environment(s) may support behavior change, CFIR provides an ideal framework for delineating multilevel factors in complex health interventions.

##### Cluster-randomized trial of the implementation strategies

**Fig. 2:**
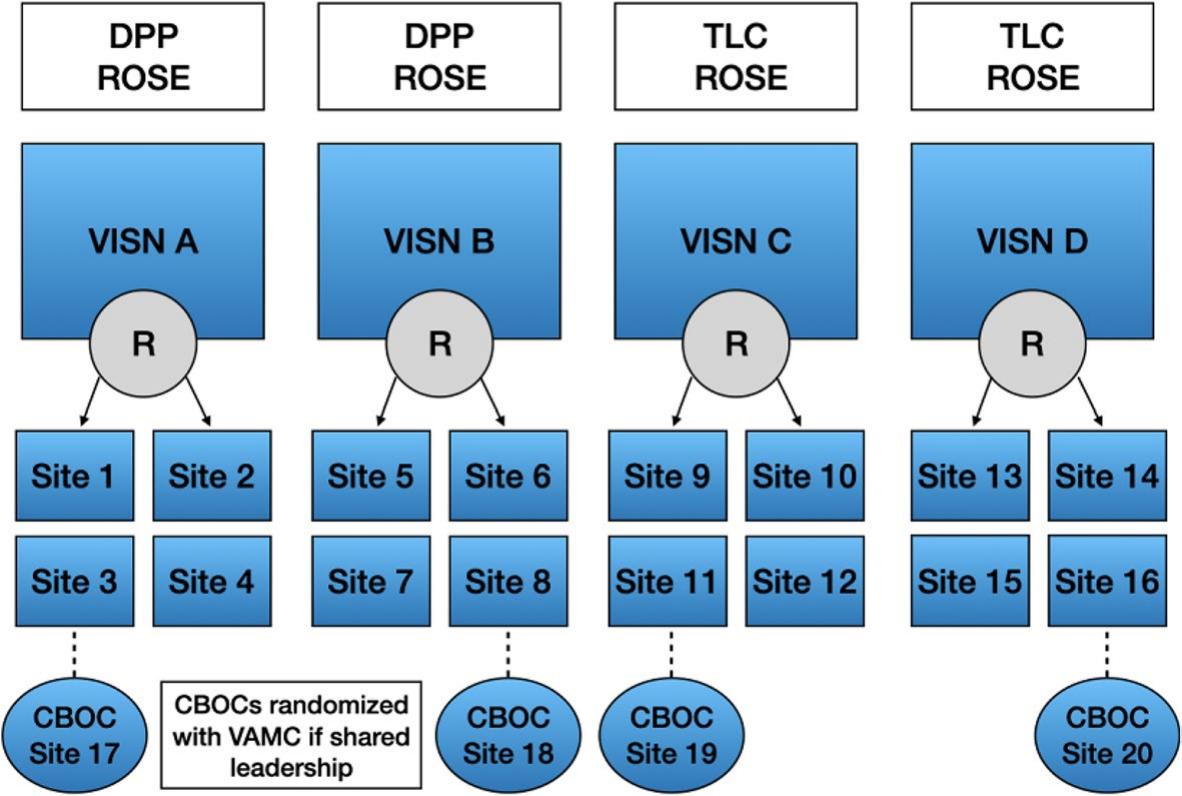
Site-level randomization

*Randomization*: For this cluster-randomized trial, we will randomize sites clustered within four VISNs to either REP or EBQI (Fig. 2). Two VISNs will implement virtual DPP and two VISNs will implement TLC. To ensure adequate number of pregnant women for ROSE, all four VISNs will have the option to implement ROSE for their pregnant patients. With our focus on increasing access to preventive services for women living in rural areas, sites that serve rural Veterans will be selectively recruited. Rural sites tend to be community-based outpatient clinics (CBOCs) as opposed to large VA medical centers (VAMCs), so at least one additional CBOC in each VISN will invited to participate. Because the same leadership team may oversee VAMC and CBOC operations, any VAMC and CBOC that share a leadership team will be randomized as a unit to avoid contamination across implementation strategies. Therefore, a total of 20 individual VA sites (VAMCs or CBOCs) will be included from four VISNs. With the balanced design, half of the sites (*N*=10) will receive REP and half will receive EBQI (*N*=10).

*Aim 2a: Data sources*—We will use VA administrative data in the Corporate Data Warehouse (CDW) via the VA Informatics and Computing Infrastructure (VINCI) platform to examine EBP access and engagement and will monitor changes and trends in VA performance measures using the Reporting, Analytics, Performance, Improvement & Deployment’s Electronic Quality Measurement (eQM) portal. Since many of the interactive algorithms available on the eQM portal are not available by site and by gender, we will use the algorithms and data available on the portal and merge patient-level data in CDW to create gender-specific performance measures focusing on diabetes, hypertension, and depression/mental health. Additional engagement and outcome data will also be collected from each EBP delivery including number of sessions/modules completed, and program-specific measures.

*Aim 2a: Measures*—These fall into four basic categories shown in Table 2. The *primary outcomes of interest* are access to and engagement in telehealth care for preventive services, measured by referrals and enrollment. We will create composite measures of the proportion of women who participate in preventive telehealth care services for each EBP (# participate/# eligible). The primary outcomes for virtual DPP include the number of women who self-register for DPP (i.e., enroll) and the number who participate in virtual DPP (i.e., complete at least one online DPP module). For TLC and ROSE, the primary outcomes include the number of provider referrals for the programs and number of women Veterans with enrollment encounters. Parallel encounter coding will be developed for ROSE with the guidance of our Office of Primary Care. *Participation and engagement* outcomes collected by EBP are based on the average number of sessions completed and behavior changes documented during the program (e.g., weight change, increase physical activity, depression screens). For virtual DPP, we will assess rates of participation and engagement according to CDC standards for DPP delivery (i.e., average number of sessions attended, proportion who completed ≥9 and ≥16 sessions, etc.). For TLC, rates of participation and engagement are at ≥3 calls and ≥8 calls based on guidance provided by VA national offices regarding meaningful levels of contact with this EBP. For ROSE, maternity care coordinators (or comparable role) and ROSE facilitators will collect data on the number of sessions completed and proportion of completion of postnatal depression screens among participants. The *primary impact performance goals* for the EBPs will be based on performance metrics for telehealth care. For virtual DPP, we will monitor site-level performance measures for participation in weight management programs (e.g., MOVE!). For TLC, we will monitor site-level changes in telehealth encounters for prevention (e.g., Whole Health). For ROSE, we will monitor site-level changes in the use of telemental health and site-level changes in number of prenatal referrals sent out of the VA. Across all EBPs, we will also monitor metrics for use of telehealth services by women Veterans living in rural areas. Secondary outcomes of interest will include *clinical outcomes and other VA performance measures* related to each EBP. For virtual DPP and TLC, we will examine trends in clinical outcomes including percent weight change over 12 and 24 months, prevalence of hypertension, pre-diabetes, and diabetes. We will also examine trends in incident type 2 diabetes (≥1 inpatient diagnosis or any combination of ≥2 within 24 months: A1C≥6.5%, or fasting glucose≥126, or random glucose ≥ 200 or 2-h 75-g oral glucose tolerance test ≥ 200, or outpatient diagnosis code, or anti-hyperglycemic medication claim except metformin) [38, 39]. The VA (HEDIS) performance measures for diabetes and hypertension focus on proportion with guideline-recommended level of control among patients with the condition. Although our project is focused on prevention, we will track these measures because EBPs focused on lifestyle behavior change could impact level of control among diabetics and patients with hypertension. For ROSE, we will examine trends in postpartum attrition from VA among pregnant women through utilization of VA health services in the 12 months post-partum.

**Table ﻿2:** Measures for Aim 2a

EBP	Primary outcome	Participation and engagement	Primary impact performance goals	Clinical outcomes and other VA performance measures
Virtual DPP	• # of eligible patients who register at the DPP site (i.e., enroll) • # of eligible patients who participate in virtual DPP and attend at least one session	• Average # of DPP sessions attended^a^ • Proportion who completed ≥3, ≥9 and ≥16 DPP sessions^a^ • Change in reported physical activity^a^ • % weight change at 12 and 24 months	Increase participation in weight management program (e.g., MOVE)	• Overweight/obesity/weight management • Prevalence of prediabetes, diabetes, and incident type 2 diabetes • HEDIS: Hypertension and Diabetes
TLC	• # of provider referrals for TLC • # of patients w/ TLC encounter (enrolled)	• Average # of TLC sessions completed^a^ • % with SMART goal^a^ • % with behavioral change (physical activity; diet: fruit, vegetable, and sugary beverage intake; stress and coping; and weight)^a^	Increase in telehealth encounters for prevention (e.g., Whole Health)	• Overweight/obesity/weight management • Prevalence of prediabetes, diabetes, and incident type 2 diabetes • HEDIS: Hypertension and Diabetes
ROSE [4]	• # of provider referrals for ROSE • # of patients w/ ROSE encounter (enrolled)	• Average # of ROSE sessions completed^a^ • Depression screening	Increase in telehealth care for mental health	• # of patients that return to VA post-partum (utilization of VA health services in the 12 months post-partum) • Mental health/depression

^a^This data will be collected as part of the EBP. All other data will come from CDW

We will include data on-site-, provider-, and patient-level variables from VA administrative data or our operations partners. For the sites, we will include facility type (VAMC or CBOC) and size, women panel size, proportion of women at site, type of comprehensive primary care model for women Veterans [4], number of designated women’s health providers, and urban/rurality. Provider characteristics will include provider type (MD or NP) and whether the provider is a designated women’s health provider. Patient-level controls will include sociodemographic characteristics, comorbidity including diabetes and cardiovascular risk, service-connected disability status, utilization, and the most common ICD-10 codes related to pregnancy status.

*Data analysis plan for Aim 2a*: We will use generalized linear models to evaluate the effectiveness of the intervention strategies for implementation of the EBPs in increasing women’s participation in telehealth-based preventive care. The models will account for clustering at the site level as well as incorporating site- and patient-level covariates outlined above. The main outcomes will include the composite measures of the proportion of women referred and enrolled in preventive telehealth care services, and the primary independent variable of interest is implementation using REP versus EBQI. We will conduct subgroup analyses by EBP to examine if the effects of the implementation strategies on proportion referred and enrolled vary by EBP (TLC or virtual DPP) as well as evaluate the effect on the *primary impact performance metrics* for telehealth care (weight management programs, Whole Health, and telemental health). In each model, we will examine the potential moderating effects of site-level characteristics (e.g., facility type, size, women’s health care program model, etc.). Adjustment for clustering will be performed using Stata v15, and we will evaluate goodness-of-fit using Mallows statistic (Cp). For DPP and TLC, we will use appropriate generalized linear models to assess weight and behavior change and examine differences by levels of participation (number of sessions completed). For ROSE, we will assess depression scores by levels of participation.

*Power calculations*: We based power calculations on the study’s basic single level of clustering sample design, where sites are randomized to receive either REP or EBQI to implement the EBPs. To detect a moderate effect size in terms of Cohen’s *D* (.26 standard deviations) between REP and EBQI, with 20 sites, and cluster adjustment (ICC=.01) with 80% power we will recruit 40 women per site (total of 800 women). We have considered all 20 sites to be separate clusters in terms of these calculations.

Aims 2b–2d are summarized in Table 3.

**Table 3:** Overview of Aims 2b–2d

Method	Timing (per QUERI Roadmap)			Intended analyses
	Pre-implementation	Implementation	Sustainment	
Site tracking log	• Meeting minutes • Templated reflections • Stages of Implementation Completion (SIC)			2b: SIC 2c: Adaptations, experiences of EBP implementation 2d: cost
Semi-structured interviews	• CFIR-based pre-implementation interview	• CFIR-based post-implementation interview		2b: SIC 2c: Adaptations, experiences of EBP implementation
Periodic reflections	• Conducted approximately monthly to document: stakeholder engagement; EBP and/or implementation strategy adaptations; inner and outer setting influences; and planning, processes, and key events.			2b: SIC 2c: Adaptations, experiences of EBP implementation

*Aim 2b: Data sources*—The *Stages of Implementation Completion (SIC)* [27] is an assessment tool developed in a large randomized trial for the purpose of comparing “progress and milestones toward successful implementation…regardless of the implementation strategy utilized” [40]. SIC allows for quantitative scoring of sites’ implementation progress, reflecting multiple components of overall implementation success. The REP and EBQI teams will gather the information necessary to populate the assessment tool for each site.

*Aim 2b: Measures*—The SIC delineates a set of eight stages and activities across three phases (Pre-Implementation, Implementation, and Sustainment, consistent with the QUERI Roadmap [35]) that are largely universal in implementation efforts (e.g., readiness planning, adherence monitoring) [41]. The SIC may be unique among implementation measures for its utility in capturing standardized yet flexible assessments suitable for comparison across sites, EBPs, and implementation strategies.

*Aim 2b: Analysis*—SIC allows for calculation of the following scores: each site’s *stage score*, which describes the ultimate stage achieved at a given site; *proportion score*, which describes the proportion of activities completed by a site within a given stage; and *duration score*, which describes the amount of time a site spends in each stage. For additional detail on mixed-method analysis of SIC data, see *Aim 2c: Analysis*.

*Aim 2c: Data sources*—Adaptations of an EBP in practice [42], how individuals and teams engage in sensemaking around the EBP and implementation process [43], and stakeholders’ observations and experiences of implementation [28, 44] are all recognized for their importance in understanding implementation and sustainment outcomes [45]. We will evaluate adaptation, sensemaking, and experiences of implementation through interviews and brief structured data collection with *multilevel key stakeholders (KS)*, defined as individuals who are “responsible for…healthcare-related decisions that can be informed by research evidence” [46]. Consistent with the Women’s Health PACT cluster randomized trial of EBQI [29], eligible roles include regional leadership, facility leadership, facility-level clinical leaders such as WH Medical Directors, facility-level Women Veteran Program Managers, and PBRN Site Leads. Expanding beyond these roles for our prevention focus will allow the inclusion of primary care, primary care mental health integration (or mental health), and Whole Health/Health Promotion Disease Prevention leaders, program managers, and providers. Individuals in these roles will be identified using publicly available information as well as lists provided by the Site Leads. Using a snowball sampling approach, individuals in the eligible roles will be asked to recommend other KS due to their women’s health expertise. Based on our prior study, we estimate 6 KS per site (120 total) and 5 KS per VISN (20 total) for a total sample at pre-implementation of *n*=140. A similar sample size is anticipated for post-implementation/pre-sustainment KS interviews. Periodic reflections (see below) will be completed with *members of the implementation team* for each EBP and site (e.g., PIs, project managers, site leads, EBQI and REP leads, etc.).

*Aim 2c: Measures*—Adaptation, sensemaking, and experiences of EBP implementation will be assessed using KS interviews, structured data collection, and periodic reflections. Semi-structured qualitative interviews will be conducted with KS during pre-implementation and post-implementation/pre-sustainment phases. Pre-implementation interviews (see Additional file 2 for a draft interview guide) will examine usual care for the relevant care condition as well as CFIR domains including inner and outer setting, perceived characteristics of the intervention, characteristics of individuals (e.g., prior training), and implementation process using semi-structured interview guides informed by CFIR online resources and our work in EMPOWER QUERI 1.0 [22] and the Women’s Health PACT trial [25, 29]. Post-implementation/pre-sustainment interviews will also assess (1) CFIR domains (e.g., perceived characteristics of the EBP) and subdomains (e.g., relative advantages, complexity, etc.) and (2) recommendations for adaptations to and/or spread of the EBP to which KS were exposed.

We have developed a site tracking log that allows facilitators and other QI leads to document site contacts and meetings; the log format includes open-ended, templated written reflections on implementation progress from the external team member’s perspective. Logs will be used to document SIC progression and date of stage completion; capture information on dose and intensity of contacts across the two implementation strategies; support development of cost estimates per strategy, per EBP, and per site; and provide qualitative data from templated reflections for analysis as described below.

Periodic reflections [28] allow for consistent documentation of key activities and other implementation phenomena and have been shown to support timely and accurate documentation of adaptations, changes to inner and outer setting, team sensemaking, and dynamic experiences of EBP implementation. Periodic reflections will be conducted approximately monthly via telephone by a member of the Implementation Core with members of the implementation team for each EBP and site, will follow the template developed for use in EMPOWER 1.0 (Additional file 3), and will last 15–60 min, depending on the amount of current activity and number of team members participating in the call (range 1–4).

*Aim 2c: Analysis plan*—All *KS interviews* will be digitally recorded and securely transmitted to an approved transcriptionist for verbatim transcription. Transcripts will be reviewed, edited for accuracy, and summarized by the qualitative team. Consistent with our team’s approach across multiple projects, matrix analysis methods [47] will be used for rapid turn-around of the results to inform implementation processes. In-depth analysis of the qualitative data will be conducted using ATLAS.ti, a qualitative data analysis software program that allows for fluid interaction of data across types and sources. Initially, a top-level codebook will be developed for the pre-implementation interviews based on CFIR constructs and the semi-structured interview guide. This codebook will be elaborated upon based on emergent themes. Interviews will be compared within each site, across sites (e.g., to compare urban and rural sites), across implementation strategies, and over time. *Periodic reflections* and other sources of qualitative data (i.e., meeting minutes, templated reflections in site tracking logs, and archival information) will also be included in the data set and will be coded separately and in relation to the interview data, with particular attention to adaptations, individual and team sensemaking, and changes over time (e.g., within CFIR domains), similar to the processes previously used by members of this team [28]. Specifically, in the pre-implementation transcripts, we will identify commonly shared knowledge, attitudes, and beliefs related to the EBPs and their foci, and anticipated barriers to and facilitators of implementation. In post-implementation interview data, we will take a summative approach to characterizing overall experiences of and perspectives on implementation, with a particular focus on expectations for sustainment of the EBPs and the implementation strategies.

It is an innovation and strength of this proposal that we will combine 2b and 2c data sources to link qualitative (via interviews, reflections, etc.) assessments of CFIR constructs with implementation and effectiveness outcomes—including SIC scores for stage, duration, and proportion—for each EBP (virtual DPP, TLC, ROSE) and implementation strategy (REP, EBQI). With the exception of recent work by Palinkas et al., relatively few studies have previously investigated associations between CFIR domains and the stages and timing of implementation milestones achieved [40]. This will enable us to investigate, for example, whether EBQI, a strategy that requires more intensive activity in the initial stages and may thus be slower to reach implementation launch, is associated with improved sustainment, given that sites will be equipped with QI tools beyond the life of the project.

*Aim 2d: Data sources*—In line with current recommendations for budget impact analysis (BIA) for single payers [48] and within VA [49], we will compare costs associated with delivery of usual care with costs for implementation and delivery of our prevention-focused telehealth interventions (virtual DPP, TLC, ROSE) in terms of impact on short-term use of downstream health care resources. For example, we will evaluate whether the cost of implementing the ROSE intervention is associated with cost savings related to a reduction in need for specialty mental health care among women Veterans during the postpartum period.

*Aim 2d: Measures*—Core requirements for BIA include estimates of eligible population, current treatment mix and associated costs, expected treatment mix post-implementation and associated costs, and estimated changes in condition-related downstream costs [50]. Cost estimates for implementation strategies will be informed by site tracking log data (e.g., implementation dose in #hours/per site) as described in *Aim 2c: Data sources*. Healthcare utilization data will be drawn from CDW, as described for *Aim 2a: Data sources*, above.

*Aim 2d: Analysis*—Analyses will be structured to examine the comparative cost and outcomes of our two implementation strategies, REP and EBQI, as follows [30]: (Cost_Intervention_ + Cost_REP_) − (Cost_Intervention_ + Cost_EBQI_) vs. Outcome_InterventionW/REP_ − Outcome_InterventionW/EBQI_. Qualitative data collected for Aim 2c will also be examined to understand the “qualitative residual” that often remains underexplored in traditional quantitative economic evaluations, aiding in improved cost estimates and understanding of how stakeholders, staff, providers, and implementation teams make sense of implementation need, impact, and cost [30]. For example, cost estimates for an EBP may need to be updated based on adaptations in routine practice reported by providers in interviews. Similarly, leadership across sites may have differing perspectives on whether a higher-intensity strategy, like EBQI, is worth investing in given observed outcomes, with implications for future sustainment and spread efforts.

EMPOWER 2.0 will evaluate the business case for REP and EBQI in implementing DPP, TLC, and ROSE by first drawing upon the Cost of Implementing New Strategies (COINS) method for identifying resources invested in implementation [51]. COINS provides a systematic way of assessing costs associated with each Stage of Implementation Completion (SIC), which provides the blueprint for the business plan [27]. In establishing our cost estimates, we will integrate data from stakeholder interviews, site tracking logs, and reflections to assess costs as they occurred: (a) at each site, (b) for each EBP, (c) for both REP and EBQI, and (d) according to each stage of the implementation effort. As a measure of implementation process, the SIC will be cross-walked with the COINS to develop template plans and timelines for future adopters. Knowing, for instance, that they need to budget for 3 months of pre-launch preparation will allow new sites to plan against their fiscal years, e.g., knowing what to budget when, and what can be spread across different fiscal cycles.

### Aim 3: Generate implementation “playbooks” for program partners that are scalable and serve as guidance for future implementation of a broader array of evidence-based women’s health programs and policies

Implementation evaluation of process and outcomes across EBPs, actively supported by the Implementation Core (see below), will contribute to the development of implementation playbooks—brief, user-friendly summations of implementation targets, processes, outcomes, and recommendations for sustainment, scale up, and spread [52]. EMPOWER 1.0 was an early QUERI champion for playbooks and has provided a laboratory for examining what kinds of information playbooks should contain for optimum utility and impact. For EMPOWER 2.0, we will work collaboratively with sites and partners to develop operations playbooks, brief “tip sheets”, and other operations-focused implementation support products as needed.

## Discussion

EMPOWER 2.0 provides a model for the conduct of a hybrid type 3 effectiveness-implementation trial comparing two implementation strategies in a large healthcare system. It is a limitation of this study that it is being conducted entirely within the VA healthcare system and thus results may not generalize; however, site- and regional-level conditions within the VA vary considerably, and we anticipate that findings will speak to common challenges in addressing the unique needs and resources of local settings.

Although this proposal was developed and submitted for funding prior to the COVID-19 pandemic, recent events have only highlighted the need for increased telehealth services and significantly expanded remote care technology integration within and outside of the VA. Pre-existing gaps faced by many healthcare systems in the uptake and reach of preventive care services have been amplified during the pandemic, exacerbated by the deferral of care for non-COVID-19 conditions [53–55]. The emphasis of this study on the implementation of preventive care services is thus an important strength, particularly as this has traditionally been an underdeveloped area of implementation science [56, 57].

As relatively few studies have compared the effectiveness of implementation strategies for achieving rollout of evidence-based interventions across large healthcare systems, the proposed rigorous comparison of clinical benefit, cost and return on investment, and time to achieving implementation associated with REP and EBQI will be of value in directly supporting evidence-based policy and resource allocation decisions. Our program will not only enhance care for women Veterans across 20 VA sites, but will also shed fundamental insights on implementation strategies to help sustain these improvements and inform broader dissemination across and beyond VA.

## Supplementary Information

**Additional file 1.** EMPOWER 2.0 Key Health Conditions and Evidence-Based Practices (EBPs).

**Additional file 2.** Sample Key Stakeholder Interview Guide.

**Additional file 3.** EMPOWER 2.0 Periodic Reflections Template.

## Data Availability

Not applicable.

## Abbreviations

BIA: Budget impact analysis
CDW: Corporate Data Warehouse
CFIR: Consolidated Framework for Implementation Research
COINS: Cost of Implementing New Strategies
DPP: Diabetes Prevention Program
EBP: Evidence-based practice
EBQI: Evidence-Based Quality Improvement
EMPOWER 2.0: Enhancing Mental and Physical Health of Women Veterans through Engagement and Retention 2.0
QUERI: Quality Enhancement and Research Initiative
PACT: Patient-aligned clinical team
REP: Replicating Effective Program
ROSE: Reach Out, Stay Strong Essentials
SIC: Stages of Implementation Completion
TLC: Telephone Lifestyle Coaching

## References

[1] Frayne SM, Phibbs CS, Saechao F (2014). Sourcebook: Women veterans in the Veterans Health Administration. Volume 3. Sociodemographics, utilization, costs of care, and health profile.

[2] Statistics NCfVAa (2017). Women Veterans Report: the past, present, and future of women veterans.

[3] deKleijn M, Lagro-Janssen AL, Canelo I, Yano EM (2015). Creating a roadmap for delivering gender-sensitive comprehensive care for women Veterans: results of a national expert panel. Med Care.

[4] Yano EM, Haskell S, Hayes P (2014). Delivery of gender-sensitive comprehensive primary care to women veterans: implications for VA Patient Aligned Care Teams. J Gen Intern Med.

[5] Bergman AA, Frankel RM, Hamilton AB, Yano EM (2015). Challenges with delivering gender-specific and comprehensive primary care to women veterans. Womens Health Issues.

[6] Chuang E, Brunner J, Mak S (2017). Challenges with implementing a patient-centered medical home model for women veterans. Womens Health Issues.

[7] Haskell SG, Mattocks K, Goulet JL (2011). The burden of illness in the first year home: do male and female VA users differ in health conditions and healthcare utilization. Womens Health Issues.

[8] Goldstein KM, Melnyk SD, Zullig LL (2014). Heart matters: gender and racial differences cardiovascular disease risk factor control among veterans. Womens Health Issues.

[9] Vimalananda VG, Biggs ML, Rosenzweig JL (2014). The influence of sex on cardiovascular outcomes associated with diabetes among older black and white adults. J Diabetes Complications.

[10] Breland JY, Phibbs CS, Hoggatt KJ (2017). The obesity epidemic in the Veterans Health Administration: prevalence among key populations of women and men veterans. J Gen Intern Med.

[11] Han JK, Yano EM, Watson KE, Ebrahimi R (2019). Cardiovascular care in women veterans. Circulation.

[12] Creech SK, Pulverman CS, Crawford JN (2021). Clinical complexity in women veterans: a systematic review of the recent evidence on mental health and physical health comorbidities. Behav Med.

[13] Hamilton AB, Frayne SM, Cordasco KM, Washington DL (2013). Factors related to attrition from VA healthcare use: findings from the National Survey of Women Veterans. J Gen Intern Med.

[14] Wagner C, Dichter ME, Mattocks K (2015). Women veterans’ pathways to and perspectives on Veterans Affairs health care. Womens Health Issues.

[15] Lehavot K, Der-Martirosian C, Simpson TL, Sadler AG, Washington DL (2013). Barriers to care for women veterans with posttraumatic stress disorder and depressive symptoms. Psychol Serv.

[16] Shipherd JC, Darling JE, Klap RS, Rose D, Yano EM (2018). Experiences in the Veterans Health Administration and impact on healthcare utilization: comparisons between LGBT and non-LGBT women veterans. LGBT Health.

[17] Klap R, Darling JE, Hamilton AB (2019). Prevalence of stranger harassment of women veterans at Veterans Affairs Medical Centers and impacts on delayed and missed care. Womens Health Issues.

[18] Friedman SA, Frayne SM, Berg E (2015). Travel time and attrition from VHA care among women veterans: how far is too far?. Med Care.

[19] Gawron LM, Pettey WBP, Redd AM, Suo Y, Turok DK, Gundlapalli AV (2019). Distance matters: geographic barriers to long acting reversible and permanent contraception for homeless women Veterans. J Soc Distress Homeless.

[20] Administration VH (2019). Veterans Health Administration modernization campaign plan.

[21] Weinert C, Cudney S, Hill WG (2008). Rural women, technology, and self-management of chronic illness. Can J Nurs Res.

[22] Hamilton AB, Farmer MM, Moin T (2017). Enhancing Mental and Physical Health of Women through Engagement and Retention (EMPOWER): a protocol for a program of research. Implement Sci.

[23] Dyer KE, Moreau JL, Finley E (2020). Tailoring an evidence-based lifestyle intervention to meet the needs of women Veterans with prediabetes. Women Health.

[24] Kilbourne AM, Neumann MS, Pincus HA, Bauer MS, Stall R (2007). Implementing evidence-based interventions in health care: application of the replicating effective programs framework. Implement Sci.

[25] Yano EM, Darling JE, Hamilton AB (2016). Cluster randomized trial of a multilevel evidence-based quality improvement approach to tailoring VA Patient Aligned Care Teams to the needs of women Veterans. Implement Sci.

[26] Landes SJ, McBain SA, Curran GM (2019). An introduction to effectiveness-implementation hybrid designs. Psychiatry Res.

[27] Saldana L (2014). The stages of implementation completion for evidence-based practice: protocol for a mixed methods study. Implement Sci.

[28] Finley EP, Huynh AK, Farmer MM (2018). Periodic reflections: a method of guided discussions for documenting implementation phenomena. BMC Med Res Methodol.

[29] Hamilton AB, Brunner J, Cain C (2017). Engaging multilevel stakeholders in an implementation trial of evidence-based quality improvement in VA women’s health primary care. Transl Behav Med.

[30] Dopp AR, Mundey P, Beasley LO, Silovsky JF, Eisenberg D (2019). Mixed-method approaches to strengthen economic evaluations in implementation research. Implement Sci.

[31] Moin T, Damschroder LJ, AuYoung M (2018). Results from a trial of an online diabetes prevention program intervention. Am J Prev Med.

[32] Damschroder LJ, Reardon CM, Sperber N, Robinson CH, Fickel JJ, Oddone EZ (2017). Implementation evaluation of the Telephone Lifestyle Coaching (TLC) program: organizational factors associated with successful implementation. Transl Behav Med.

[33] Zlotnick C, Tzilos G, Miller I, Seifer R, Stout R (2016). Randomized controlled trial to prevent postpartum depression in mothers on public assistance. J Affect Disord.

[34] Huynh AK, Hamilton AB, Farmer MM (2018). A pragmatic approach to guide implementation evaluation research: strategy mapping for complex interventions. Front Public Health.

[35] Kilbourne AM, Goodrich DE, Miake-Lye I, Braganza MZ, Bowersox NW (2019). Quality enhancement research initiative implementation roadmap: toward sustainability of evidence-based practices in a learning health system. Med Care.

[36] Damschroder LJ, Aron DC, Keith RE, Kirsh SR, Alexander JA, Lowery JC (2009). Fostering implementation of health services research findings into practice: a consolidated framework for advancing implementation science. Implement Sci.

[37] Kirk MA, Kelley C, Yankey N, Birken SA, Abadie B, Damschroder L (2016). A systematic review of the use of the Consolidated Framework for Implementation Research. Implement Sci.

[38] Nichols GA, Schroeder EB, Karter AJ (2015). Trends in diabetes incidence among 7 million insured adults, 2006-2011: the SUPREME-DM project. Am J Epidemiol.

[39] Lipscombe LL, Hwee J, Webster L, Shah BR, Booth GL, Tu K (2018). Identifying diabetes cases from administrative data: a population-based validation study. BMC Health Serv Res.

[40] Palinkas LA, Campbell M, Saldana L (2018). Agency leaders’ assessments of feasibility and desirability of implementation of evidence-based practices in youth-serving organizations using the stages of implementation completion. Front Public Health.

[41] Chamberlain P, Brown CH, Saldana L (2011). Observational measure of implementation progress in community based settings: the Stages of Implementation Completion (SIC). Implement Sci.

[42] Stirman SW, Baumann AA, Miller CJ (2019). The FRAME: an expanded framework for reporting adaptations and modifications to evidence-based interventions. Implement Sci.

[43] Stensaker I, Falkenberg J, Grønhaug K (2008). Implementation activities and organizational sensemaking. J Appl Behav Sci.

[44] George AS, Branchini C (2017). Principles and processes behind promoting awareness of rights for quality maternal care services: a synthesis of stakeholder experiences and implementation factors. BMC Pregnancy Childbirth.

[45] Proctor E, Silmere H, Raghavan R (2011). Outcomes for implementation research: conceptual distinctions, measurement challenges, and research agenda. Adm Policy Ment Health.

[46] Concannon TW, Meissner P, Grunbaum JA (2012). A new taxonomy for stakeholder engagement in patient-centered outcomes research. J Gen Intern Med.

[47] Averill JB (2002). Matrix analysis as a complementary analytic strategy in qualitative inquiry. Qual Health Res.

[48] Eisman AB, Kilbourne AM, Dopp AR, Saldana L, Eisenberg D. Economic evaluation in implementation science: making the business case for implementation strategies. Psychiatry Res. 2019;283. 10.1016/j.psychres.2019.06.008 PMC6898762 31202612

[49] Wagner TaS A (2019). Economic and budget impact analysis.

[50] Sullivan SD, Mauskopf JA, Augustovski F (2014). Budget impact analysis-principles of good practice: report of the ISPOR 2012 Budget Impact Analysis Good Practice II Task Force. Value Health.

[51] Saldana L, Chamberlain P, Bradford WD, Campbell M, Landsverk J (2014). The Cost of Implementing New Strategies (COINS): a method for mapping implementation resources using the stages of implementation completion. Child Youth Serv Rev.

[52] Sharp A, Nguyen H, Hahn E (2014). The just do it playbook for implementation science.

[53] DeJong C, Katz MH, Covinsky K (2021). Deferral of care for serious non-COVID-19 conditions: a hidden harm of COVID-19. JAMA Intern Med.

[54] Blecker S, Jones SA, Petrilli CM (2021). Hospitalizations for chronic disease and acute conditions in the time of COVID-19. JAMA Intern Med.

[55] Wright A, Salazar A, Mirica M, Volk LA, Schiff GD (2020). The invisible epidemic: neglected chronic disease management during COVID-19. J Gen Intern Med.

[56] Wiggers J, McElwaine K, Freund M (2017). Increasing the provision of preventive care by community healthcare services: a stepped wedge implementation trial. Implement Sci.

[57] McElwaine KM, Freund M, Campbell EM, Bartlem KM, Wye PM, Wiggers JH (2016). Systematic review of interventions to increase the delivery of preventive care by primary care nurses and allied health clinicians. Implement Sci.

