# Supplementary material for: Enhancing Mental and Physical Health of Women through Engagement and Retention (EMPOWER) 2.0 QUERI: study protocol for a cluster-randomized hybrid type 3 effectiveness-implementation trial
Source: Implement Sci Commun. 2023 Mar 8;4:23. doi: 10.1186/s43058-022-00389-w (PMC9994412; doi:10.1186/s43058-022-00389-w)
Supplement: Supplementary file 1 — Additional file 1. EMPOWER 2.0 Key Health Conditions and Evidence-Based Practices (EBPs). [file 43058_2022_389_MOESM1_ESM.docx]

**Additional file 1 – EMPOWER 2.0 Key Health Conditions and Evidence-Based Practices (EBPs)**

***Key Health Conditions for Women Veterans***

In a recent systematic review of VA women’s health research, Danan and colleagues found that only three or fewer of 440 articles reviewed addressed each of the common chronic diseases: diabetes, hypertension, depression, or anxiety.^1^ For example, they found no studies with a primary focus on hypertension in women Veterans, and they note that mental health topics encountered in primary care, such as postpartum depression, “were largely absent from the literature.” Below we briefly review why each of our key health conditions of interest represent critical gaps in women Veterans’ health research to date.

*Type 2 diabetes* is more prevalent among Veterans than civilians,^2^ affecting 25% of Veterans receiving care in VA, and is associated with significant morbidity and mortality. Prediabetes is even more prevalent than diabetes: an estimated 33.9% of U.S. adults aged 18 years or older (84.1 million people) had prediabetes in 2015. Among adults with prediabetes, only 11.6% reported being told by a health professional that they had this condition.^3^ Adults with prediabetes are at increased risk of developing diabetes and diabetic complications such as retinopathy. However, numerous randomized controlled trials, including the Diabetes Prevention Program (DPP) study, have shown that intensive lifestyle interventions can reduce the risk of incident diabetes by as much as 58% among overweight/obese adults with prediabetes.^4^ Even modest amounts of weight loss with DPP, such as 2.2lbs, is associated with as much as 16% relative diabetes risk reduction over three years of follow-up.^5^ In addition, DPP-based intensive lifestyle interventions have been shown to be cost-effective and even cost saving on the order of $2650 per participant over 15 months of follow-up.^6^ Demonstration of both clinical and cost-effectiveness of DPP has prompted CMS and many large US insurance providers to include DPP as a covered benefit. Ensuring that all at-risk adults in the US have access to DPP is also prioritized by the CDC and all national care guidelines.^7^ Women Veterans with diabetes tend to have worse outcomes than their male counterparts, emphasizing the need for evidence-based preventive services. However, women Veterans are also more reluctant to engage in diabetes prevention and weight management services in VA.

*Cardiovascular disease (CVD)* is the number one cause of death in women, with 44 million women in the United States either living with or at risk for heart disease.^8^ However, women have limited understanding of their CV morbidity and mortality risks.^9^ Moreover, women’s CV risk factors are not as well controlled as men’s, including cholesterol level, blood pressure, and diabetes intermediate outcome measures.^10^ CV guidelines support directed lipid treatment and more personalized evaluations by providers, in addition to educating patients about their risks and strongly recommending lifestyle changes (i.e., physical activity, diet, and behavioral counseling).^11^ However, numerous provider barriers to CV risk management have been identified, including lack of time, lack of awareness and understanding of the latest CVD prevention guidelines, difficulty accessing relevant electronic medical record (EMR) data at the point of care, low self-efficacy to counsel patients in behavioral change, habit or inertia, fragmentation of care, and perceptions of low patient interest or capacity to follow-through on recommendations.^12^ At the patient level, perception of personal CV risk remains limited but risk reduction interventions have been successful. For example, gender- and non-gender-specific smoking cessation programs are effective at helping women abstain from smoking.^13^ However, CV risk reduction challenges persist with weight management programs.^14^ In VA, CV risk reduction and management remains a significant focus despite rates of prevention at or above national levels according to HEDIS and AHRQ.^10^ The combination of disparities and gender-specific CV risk factors, recognized contribution of obesity and inactivity, and limited knowledge explaining barriers in risk factor reduction for women Veterans suggest an urgent need for solutions to improve CV risks and outcomes.

*Perinatal depression* is a common complication affecting one in seven women and is associated with myriad adverse outcomes for mother and child, including: increased risk of suicidal ideation in mothers, and long-term mental and behavioral health problems in children.^15^ Perinatal depression, which includes the onset of major or minor depressive episodes during pregnancy or first 12-months post-partum,^16^ has numerous risk factors including a history of depression, current depressive symptoms, a history of physical and sexual abuse, unwanted and unplanned pregnancy, intimate partner violence, stressful life events, pregestational or gestational diabetes, and pregnancy complications.^17^ Other risk factors include low socioeconomic status, adolescent parenthood, and lack of social support. Women Veterans experience additional unique stressors which may further increase the risk of perinatal depression and/or suicidal ideation, including: combat-related exposures,^18^ military sexual trauma (defined as sexual assault or harassment occurring during military service),^19^ and PTSD.^20^ While perinatal depression has been well-studied in the general population, studies in women Veterans are only recently emerging. A study of 501 pregnant women Veterans found 28% had clinically significant depressive symptoms, exceeding the high-end prevalence estimates of perinatal depression among pregnant civilian women.^21^ Women Veterans who utilize VA prenatal care benefits have higher rates of self-reported depression, current depressive and PTSD symptoms, and PTSD compared with pregnant women Veterans who use other insurance methods during pregnancy.^22,23^ Taken together, these factors speak to the urgent need for evidence-based interventions to prevent perinatal depression in women Veterans receiving VA care.

***Evidence-Based Practices (EBPs) to Prevent Type 2 Diabetes, CVD, and Perinatal Depression***

**Virtual Diabetes Prevention Program (DPP):** DPP, an evidence-based lifestyle intervention emphasizing moderate weight loss, diet, and >150 minutes per week of physical activity, has been shown in RCTs to prevent and/or delay progression to diabetes.^4^ Virtual DPP, which includes small, closed virtual groups and human coaches, is recommended by the CDC, meets US Preventive Services Task recommendations for care of persons at risk for diabetes, and adds to the menu of available VA weight management services. Our prior DPP work within VA includes over seven years of experience with a commercially-available virtual DPP-based group lifestyle intervention, known as *Prevent (*developed by Omada Health), which utilizes a CDC-approved DPP curriculum, integrates educational modules, health coaching, and tracking tools and leverages social media principles using a small group format.^24^ All participants are provided with a 12-month membership to *Prevent*. Modules are released weekly and participants choose when to log in and for how long and are supported by a certified professional female health coach. In our prior VA studies, Veterans enrolled in virtual and in-person DPP had similar weight loss but both groups lost significantly more weight as compared to MOVE! at 12-months follow-up.^25^ Within EMPOWER 1.0 we evaluated gender-tailored DPP in a QI project at one VA site. Among 302 women Veterans who met eligibility, less than one-third were aware they had prediabetes and only 16% (n=49) had previously participated in a lifestyle change program; the majority favored virtual (74%) over in-person (24%) DPP. *Higher than anticipated patient demand for DPP resulted in expansion to serve up to 120 women rather than 40 initially planned.* Between June 2016 and March 2017, a total of N=119 women Veterans enrolled in DPP (n=51 in-person, n=68 virtual). As compared to women Veterans enrolled in in-person DPP, those enrolled in virtual DPP had higher rates of participation (66% vs. 27% completed ≥9 sessions/modules) and greater weight loss (7.1 vs 3.1 lbs.). These findings aligned with our prior VA DPP work indicating that women Veterans viewed DPP as an appealing way of initiating lifestyle changes that made them feel accountable in achieving daily goals.^26^

**Telephone Lifestyle Coaching Program (TLC)**: Developed by the National Center for Disease Prevention and Health Promotion (NCP), TLC is a theory-guided program involving telephone-based, individual-level, personalized health coaching focused on disease prevention and wellness.^27^ TLC was developed using elements of a variety of behavioral health approaches and theories including motivational interviewing, cognitive behavioral therapy, social change theory, and components of mindfulness practices. Experienced coaches who receive TLC training work with Veterans to set goals and a tailored action plan in health areas such as weight management, nutrition, stress management, and smoking cessation, among others. In 2010, a pilot roll-out to evaluate TLC began at VHA; all VHA medical facilities were invited to participate and 24 medical centers enrolled. The aim was to improve six health behaviors: “Be Tobacco Free, Eat Wisely, Be Physically Active, Strive for a Healthy Weight, Limit Alcohol, and Manage Stress.” The telephone coaching was accessible during and outside of normal business hours, 6 days a week in all time zones; this was particularly helpful for the 50% of participants who reported travelling more than 40 minutes to receive care at their closest VHA. TLC coaches were required to document interactions with patients in the EHR at the time of initial enrollment, 45 days post-enrollment, and 6 months post-enrollment. Of the 9,357 Vets who were referred, 57% enrolled, 17% of whom were women. The most common goals during the first coaching call were tobacco cessation and weight loss. At the 6-month mark, 33% of participants reported having a statistically significant weight loss of 5% or more. In addition to a variety of other positive clinical and behavioral changes among participants, both participants and staff reported high satisfaction with the TLC approach overall. 95% of Vets would recommend the program to others and 90% of staff would recommend it for their facility. The outstanding results of this pilot roll-out led to further roll-out across 20 sites in FY19. TLC has yet to be gender-tailored; both NCP and WHS are supportive of concentrated efforts to increase uptake among women Veterans and to improve women Veterans’ cardiovascular health. We will draw on our experience of successfully implementing the CV Toolkit in EMPOWER QUERI 1.0 to support implementation of TLC for women. TLC implementation for women Veterans builds on the across-site requests from patients and providers at all four sites of CV toolkit in EMPOWER 1.0 asking for online or telephone options for coaching to set CV SMART goals.

**Reach Out, stay Strong, Essentials (ROSE):** ROSE is an evidence-based intervention for preventing post-partum depression (PPD) among racially and ethnically diverse low-income women at high risk for PPD.^28^ The intervention teaches women interpersonal psychotherapy (IPT) skills to improve communication and bolster social support and is administered in small groups at outpatient clinics providing prenatal care. ROSE includes four 90-mininute group sessions, plus a 50-min post-delivery “booster” session to reinforce skills learned in previous group sessions. Each session includes easy-to-understand handouts and homework. The effectiveness of the ROSE intervention has been studied in five randomized controlled trials (RCTs).^29-33^ The samples in these studies include low-income women, African-American women, adolescents, and women at high risk for postpartum depression. The primary outcome measure in all trials was occurrence of major depressive disorder or episode in the post-partum period. In a systematic review of preventive interventions for perinatal depression in pregnant and post-partum women, the US Preventive Services Task Force found that ROSE and one other intervention reduced the relative risk of perinatal depression by 53 and 50%, respectively. ROSE is now part of an on-going trial to evaluate its sustainment in 90 outpatient clinics providing prenatal care to women receiving public assistance, but none of the clinics include VA facilities.^28^

References

1. Danan ER, Krebs EE, Ensrud K, et al. An Evidence Map of the Women Veterans' Health Research Literature (2008-2015). *Journal of general internal medicine.* 2017;32(12):1359-1376.

2. Liu Y, Sayam S, Shao X, et al. Peer Reviewed: Prevalence of and Trends in Diabetes Among Veterans, United States, 2005–2014. *Preventing chronic disease.* 2017;14.

3. Control CfD. Estimates of Diabetes and Its Burden in the United States. *National Diabetes Statistics Report, 2017.* 2017.

4. Knowler WC, Barrett-Connor E, Fowler SE, et al. Reduction in the incidence of type 2 diabetes with lifestyle intervention or metformin. *The New England journal of medicine.* 2002;346(6):393-403.

5. Herman WH, Edelstein SL, Ratner RE, et al. Effectiveness and cost-effectiveness of diabetes prevention among adherent participants. *Am J Manag Care.* 2013;19(3):194-202.

6. Khan T, Tsipas S, Wozniak G. Medical Care Expenditures for Individuals with Prediabetes: The Potential Cost Savings in Reducing the Risk of Developing Diabetes. *Popul Health Manag.* 2017;20(5):389-396.

7. Association AD. Introduction: standards of medical care in diabetes—2018. In: Am Diabetes Assoc; 2018.

8. Raeisi-Giglou P, Volgman AS, Patel H, Campbell S, Villablanca A, Hsich E. Advances in cardiovascular health in women over the past decade: Guideline recommendations for practice. *Journal of Women's Health.* 2018;27(2):128-139.

9. Mosca L, Hammond G, Mochari-Greenberger H, Towfighi A, Albert MA. Fifteen-year trends in awareness of heart disease in women: results of a 2012 American Heart Association national survey. *Circulation.* 2013;127(11):1254-1263, e1251-1229.

10. Bird CE, Manocchia M, Tomblin B, et al. Mapping the gaps: Gender differences in preventive cardiovascular care among managed care members in four metropolitan areas. *Women's Health Issues.* 2018;28(5):446-455.

11. Stone NJ, Robinson JG, Lichtenstein AH, et al. 2013 ACC/AHA guideline on the treatment of blood cholesterol to reduce atherosclerotic cardiovascular risk in adults: a report of the American College of Cardiology/American Heart Association Task Force on Practice Guidelines. *Journal of the American College of Cardiology.* 2014;63(25 Pt B):2889-2934.

12. Crosson JC, Heisler M, Subramanian U, et al. Physicians' perceptions of barriers to cardiovascular disease risk factor control among patients with diabetes: results from the translating research into action for diabetes (TRIAD) study. *Journal of the American Board of Family Medicine : JABFM.* 2010;23(2):171-178.

13. Turnbull F, Woodward M, Neal B, et al. Do men and women respond differently to blood pressure-lowering treatment? Results of prospectively designed overviews of randomized trials. *European heart journal.* 2008;29(21):2669-2680.

14. Church TS, Earnest CP, Skinner JS, Blair SN. Effects of different doses of physical activity on cardiorespiratory fitness among sedentary, overweight or obese postmenopausal women with elevated blood pressure: a randomized controlled trial. *Jama.* 2007;297(19):2081-2091.

15. Santos IS, Matijasevich A, Barros AJ, Barros FC. Antenatal and postnatal maternal mood symptoms and psychiatric disorders in pre-school children from the 2004 Pelotas Birth Cohort. *Journal of affective disorders.* 2014;164:112-117.

16. Gavin NI, Gaynes BN, Lohr KN, Meltzer-Brody S, Gartlehner G, Swinson T. Perinatal depression: a systematic review of prevalence and incidence. *Obstetrics and gynecology.* 2005;106(5 Pt 1):1071-1083.

17. Kleine I. Interventions to prevent perinatal depression: US Preventive Services Task Force Recommendation Statement. *Archives of disease in childhood Education and practice edition.* 2019.

18. Nguyen S, Leardmann CA, Smith B, et al. Is military deployment a risk factor for maternal depression? *Journal of women's health (2002).* 2013;22(1):9-18.

19. Gross GM, Kroll-Desrosiers A, Mattocks K. A Longitudinal Investigation of Military Sexual Trauma and Perinatal Depression. *Journal of women's health (2002).* 2019.

20. Shivakumar G, Anderson EH, Suris AM. Managing posttraumatic stress disorder and major depression in women veterans during the perinatal period. *Journal of women's health (2002).* 2015;24(1):18-22.

21. Kroll-Desrosiers AR, Crawford SL, Moore Simas TA, Clark MA, Bastian LA, Mattocks KM. Rates and Correlates of Depression Symptoms in a Sample of Pregnant Veterans Receiving Veterans Health Administration Care. *Women's health issues : official publication of the Jacobs Institute of Women's Health.* 2019;29(4):333-340.

22. Katon JG, Gerber MR, Nillni YI, Patton EW. Consequences of Military Sexual Trauma for Perinatal Mental Health: How Do We Improve Care for Pregnant Veterans with a History of Sexual Trauma? *Journal of women's health (2002).* 2019.

23. Mattocks KM, Skanderson M, Goulet JL, et al. Pregnancy and mental health among women veterans returning from Iraq and Afghanistan. *Journal of women's health (2002).* 2010;19(12):2159-2166.

24. Sepah SC, Jiang L, Peters AL. Translating the Diabetes Prevention Program into an Online Social Network: Validation against CDC Standards. *Diabetes Educ.* 2014;40(4):435-443.

25. Moin T, Damschroder LJ, AuYoung M, et al. Results From a Trial of an Online Diabetes Prevention Program Intervention. *American journal of preventive medicine.* 2018;55(5):583-591.

26. Moin T, Bean-Mayberry, B., Zuchowski, J., Dyer, K., Huynh, A., Farmer, M., Finley, E., Hamilton, A. Extending the Reach of DPP Interventions for Women with Prediabetes: Tailoring to Enhance Translation in Primary Care. *Poster presentation at the 2018 Society for Behavioral Medicine Annual Meeting.* 2018.

27. Prevention NCfHPaD. National Telephone Lifestyle Coaching Pilot Evaluation Report. 2014.

28. Johnson JE, Wiltsey-Stirman S, Sikorskii A, et al. Protocol for the ROSE sustainment (ROSES) study, a sequential multiple assignment randomized trial to determine the minimum necessary intervention to maintain a postpartum depression prevention program in prenatal clinics serving low-income women. *Implementation science : IS.* 2018;13(1):115.

29. Zlotnick C, Johnson SL, Miller IW, Pearlstein T, Howard M. Postpartum depression in women receiving public assistance: pilot study of an interpersonal-therapy-oriented group intervention. *The American journal of psychiatry.* 2001;158(4):638-640.

30. Zlotnick C, Miller IW, Pearlstein T, Howard M, Sweeney P. A preventive intervention for pregnant women on public assistance at risk for postpartum depression. *The American journal of psychiatry.* 2006;163(8):1443-1445.

31. Zlotnick C, Tzilos G, Miller I, Seifer R, Stout R. Randomized controlled trial to prevent postpartum depression in mothers on public assistance. *Journal of affective disorders.* 2016;189:263-268.

32. Crockett K, Zlotnick C, Davis M, Payne N, Washington R. A depression preventive intervention for rural low-income African-American pregnant women at risk for postpartum depression. *Archives of women's mental health.* 2008;11(5-6):319-325.

33. Phipps MG, Raker CA, Ware CF, Zlotnick C. Randomized controlled trial to prevent postpartum depression in adolescent mothers. *American journal of obstetrics and gynecology.* 2013;208(3):192.e191-196.
