# Supplementary material for: Enhancing Mental and Physical Health of Women through Engagement and Retention (EMPOWER) 2.0 QUERI: study protocol for a cluster-randomized hybrid type 3 effectiveness-implementation trial
Source: Implement Sci Commun. 2023 Mar 8;4:23. doi: 10.1186/s43058-022-00389-w (PMC9994412; doi:10.1186/s43058-022-00389-w)
Supplement: Supplementary file 2 — Additional file 2. Sample Key Stakeholder Interview Guide. [file 43058_2022_389_MOESM2_ESM.docx]

**Additional file 2 – Sample Key Stakeholder Interview Guide**

EMPOWER 2.0 QUERI

Pre-Implementation Key Stakeholder Interview Guide

**EBP: Telephone Lifestyle Coaching (TLC)**

Stakeholders: WVPM, PC Director, WH Director, PCPs who see women, PACT RN, LVN, health tech, health coach/HPDP, nutrition, pharmacy, front office staff (MSAs)

*Introduce self, and if applicable, introduce additional interviewer(s) by name.*

Hi my name is ___________________ *(if applicable)* and this is/these are my colleague(s) _______________. S/he is/They are with me today to help ensure that I cover all the bases and to take notes.

Thank you for taking the time to meet with us today.

Before we begin, let me review some general information. This interview is part of a implementation effort focused on care for women Veterans with cardiovascular risks, and is expected to last no more than one hour.

This interview is entirely voluntary. You are free to skip questions, or stop or postpone this interview at any time.

To protect privacy, throughout this interview I will not refer to you or any of your colleagues by name. It would be helpful if you could also refer to colleagues by title rather than name. If you forget and do so, we will just redact them when we make the transcript.

Do you have any questions about the interview or about your participation?

If you have any questions after the interview, the PI’s contact information is included in the Q&A Addendum we sent you. *(Provide your own card if you like. If they don’t have the Q&A, offer to send again).*

I’m now going to ask if you agree to be recorded. May I record this interview session?

[If Yes] Thank you. I will now turn on the recorder and re-ask this question of you to record your oral permission to record. [Turn on Recorder] This interview is being recorded. I am asking your oral permission to be recorded. Do you grant me your permission to record this interview session? [pause for “Yes” answer] As stated before in our earlier conversation, you can ask me to pause or turn off the recorder at any time.

[If No] OK, I will not be recording this session but only taking notes of our conversation.

[If recording] This is code number XXXXXX, and the date is XXXXXXX.

CFIR Domain: Intervention Characteristics

1. Have you heard about the Telephone Lifestyle Coaching or TLC intervention? If so, what do you know about it? (Intervention Source)
   - If have not heard of TLC, explain as follows:

The **Telephone Lifestyle Coaching Program (TLC)** was developed by VA’s National Center for Disease Prevention and Health Promotion (NCP), and provides telephone-based personalized health coaching to individual Veterans, focused around disease prevention and wellness. Experienced coaches who receive TLC training work with Veterans to set goals and a tailored action plan in health areas such as weight management, nutrition, stress management, and smoking cessation, among others.

1. Why is TLC being implemented in your setting? (Intervention Source)
2. How does TLC compare to other similar existing programs in your setting? (Relative Advantage)
   - What advantages or disadvantages does TLC have compared to existing programs?
3. What kinds of changes or alterations do you think you will need to make to TLC so it will work well in your setting? (Adaptability)
   - Do you think you will be able to make these changes? Why or why not?

CFIR Domain: Outer Setting

1. How well do you think TLC will meet the needs of the women Veterans served by your organization? (Patient Needs & Resources)
   - In what ways will TLC meet their needs? E.g. improved access to services? Reduced wait times? Help with self-management? Reduced travel time and expense?

1. How do you think the Veterans served by your organization will respond to TLC? (Patient Needs & Resources)
2. What kind of performance measures, policies, regulations, or guidelines influenced the decision to implement TLC? (External Policies & Incentives)
   - How will TLC affect your organization's ability to meet these measures, policies, regulations, or guidelines?

CFIR Domain: Inner Setting

1. What do you expect to get in the way of implementing TLC? Are there changes that will be needed locally to accommodate TLC? (Barriers; Structural Characteristics)
   - Changes in scope of practice? Changes in formal policies? Changes in information systems or electronic records systems? Other?
2. What is the general level of receptivity in your organization to implementing TLC? (Implementation Climate) Why?
3. To what extent might the implementation take a backseat to other high-priority initiatives going on now? (Relative Priority) What kinds of high-priority initiatives or activities are already happening in your setting?
4. Do you expect to have sufficient resources to implement and administer TLC? (Available Resources) Why or why not?

CFIR Domain: Characteristics of Individuals

1. How do you feel about the plan to implement TLC in your setting? (Knowledge & Beliefs)
   - Do you have any feelings of anticipation? Stress? Enthusiasm? Why?

CFIR Domain: Process

1. Will someone (or a team) outside your organization be helping you with implementing TLC? (External Change Agents) Can you tell me more about that?
2. Who are the key individuals to get on board with TLC? (Key Stakeholders)
   - To encourage individuals to use TLC? To help with implementation?
3. How will you know if TLC implementation has been successful? (Reflecting & Evaluating)

Do you have any other thoughts about TLC or improvement efforts that you would like to share today?

Thank you for your time!
