# Supplementary material for: Enhancing Mental and Physical Health of Women through Engagement and Retention (EMPOWER) 2.0 QUERI: study protocol for a cluster-randomized hybrid type 3 effectiveness-implementation trial
Source: Implement Sci Commun. 2023 Mar 8;4:23. doi: 10.1186/s43058-022-00389-w (PMC9994412; doi:10.1186/s43058-022-00389-w)
Supplement: Supplementary file 3 — Additional file 3. EMPOWER 2.0 Periodic Reflections Template. [file 43058_2022_389_MOESM3_ESM.docx]

**Additional file 3 – EMPOWER 2.0 Periodic Reflections Template**

**Date:**

Goals and Focus: These reflections are intended to provide an opportunity to check in regularly about how implementation efforts are going. Our main goal is to take a few minutes to discuss, document, and reflect on key activities, events, and changes occurring over the course of implementation.

**Names:**

**Roles:**

**Status update: What are the current main activities for the project? How is it going?**

**Have there been any changes to the intervention or implementation plan in the past month? Have sites encountered any particular challenges?**

**Have there been any stakeholder engagement efforts in the past month?**

**Have you seen any recent changes in the local or national environment that you think may have impact for the study?**

**What challenges have arisen in using the REP or EBQI implementation strategies?**

**Quick check: What’s going well right now? What’s not going so well?**

**What are the next steps going forward?**

**Other useful areas for discussion:**

- Barriers/concerns that have arisen recently? What solutions have been tried? How is that going?
- Who have been the key people involved in recent activities, efforts, and discussions? What have been their primary concerns, hopes, and/or suggestions?
- Have there been any surprises lately, or unexpected events?
- What lessons have been learned?
- Remaining thoughts, observations, or concerns?
